# Emerging ideas to better understand and prevent stillbirths

**DOI:** 10.1186/1471-2393-12-S1-A1

**Published:** 2012-08-28

**Authors:** Edwin A Mitchell

**Affiliations:** 1 University of Auckland, New Zealand

## 

It is estimated that over 3.6 million babies are stillborn each year [1]. Although the majority of these occur in low-income countries, stillbirth continues to place a significant burden on maternity services in high-income settings where approximately 1 in 200 infants born after 24 weeks gestation is stillborn [1]. Despite advances in ultrasound detection of lethal fetal anomalies and widespread access to antenatal care, the stillbirth rate in many high-income countries has not decreased in over two decades. Stillbirth remains an enigma, in part due to lack of research investigation, but also due to a failure to accurately identify causes and understand how they lead to stillbirth. Recent meta-analyses resulting from international collaborations have highlighted the need to expand the understanding of stillbirth.

To this end, a meeting, the Stillbirth Summit, presented by the Star Legacy Foundation and supported by various organizations, was held in October 2011, Minneapolis, MN (USA) to discuss emerging ideas in the field of stillbirth research and management. In particular the focus was on the placenta, cord, infection and inflammation, reduced fetal movements and maternal sleep. Attendees were invited researchers, stillbirth advocates and parents.

The aim of this supplement is to briefly summarise the scientific aspects of the meeting.
